# Study on Single Event Effect Simulation in T-Shaped Gate Tunneling Field-Effect Transistors

**DOI:** 10.3390/mi12060609

**Published:** 2021-05-24

**Authors:** Chen Chong, Hongxia Liu, Shulong Wang, Shupeng Chen, Haiwu Xie

**Affiliations:** Key Laboratory for Wide-Band Gap Semiconductor Materials and Devices of Education, The School of Microelectronics, Xidian University, Xi’an 710071, China; 18829029042@163.com (C.C.); chenshupeng999@126.com (S.C.); xiehaiwu.love@163.com (H.X.)

**Keywords:** tunneling field-effect transistors (TFETS), single-event effect (SEE), T-shaped gate tunneling field-effect transistors (TGTFET), fully depleted silicon on insulator (FDSOI), linear energy transfer (LET)

## Abstract

Tunneling field-effect transistors (TFETS) can reduce the subthreshold swing (SS) to below 60 mV/decade due to their conduction mechanism with band-to-band tunneling (BTBT), thereby reducing power consumption. T-shaped gate tunneling field-effect transistors (TGTFET) adapt double source and T-shaped gates to enhance the on-state current and to generate the tunneling probability. In this paper, TGTFET subjected to heavy-ion irradiation is studied by technology computer-aided design (TCAD) simulation for the first time. The results show that as the drain bias and linear energy transfer (LET) increase, the transient current and collected charge also increase. When LET = 100 MeV·cm^2^/mg and V_d_ = 0.5 V, the transient current of TGTFET is as high as 10.63 mA, which is much larger than the on-state current. This means that TGTFET is more sensitive to single-event effect (SEE) than FDSOI. By simulating a heavy-ion strike on different locations in TGTFET, the tunneling junction is the most sensitive region of SEE. This provides guidance for future research on the antiradiation application of TFET-based devices.

## 1. Introduction

In nanoelectronic circuits, power consumption has become a major obstacle to development. Due to the limitation of the SS and the nonscalability of the threshold voltage (V_t_), it is not feasible to reduce the power consumption only by changing the power supply voltage [1,2,3,4]. Due to the BTBT conduction mechanism, the TFET device overcomes the limitation of thermal electron emission and has a SS that can be less than 60 mV/decade [5,6,7,8,9,10]. Meanwhile, TFET can also reduce the short channel effect [11,12]. However, there are the three problems of bipolar effect, low on current, and large Miller capacitance, which hinder the development of TFET. Therefore, many methods have been established to improve the performance of TFET, such as the double gate structure [13], L-shaped channel TFET (LTFET) [14], U-shaped channel TFET (UTFET) [15], heterojunction structure [16], and heterogate structure [17]. The T-shaped gate tunneling field-effect transistors (TGTFET) device in this paper is a vertical TFET with a dual source T-shaped gate. It has the characteristics of a large on-state current and tunneling region [18].

In general, when irradiating the device, the total ionization dose (TID) effect and SEE are mainly studied [19,20]. Recently, TID on the electrical characteristics of heterojunction NTFETs has been studied. Studies have shown that under the same TID conditions, P-well TFETs are more robust than P-well-less TFETs [21]. This laid the foundation for the future manufacture of the heterojunction TFET’s anti-irradiation reinforcement process. Meanwhile, studies have shown that a fully depleted structure is more suitable for reducing the radiation sensitivity of future silicon-on-insulator (SOI) technology because it reduces the sensitivity of bipolar amplification and the drain region simultaneously [22]. Recently, the single-event effect (SEE) in traditional planar TFET, pocket-based planar TFET, and L-shaped TFETs has been studied. In these three TFET structures, from the values of the collected and deposited charges, it can be inferred that the antiradiation performance of the L-shaped TFET is better than that of the other two structures [23]. However, it does not specify its physical mechanism. The impact of heavy-ion impact on LTFET and FDSOI has been studied [24]. Research shows that LTFET can eliminate the bipolar amplification effect, and the tunneling junction is a SEE-sensitive region. However, large LETs are not considered.

TGTFET has development potential in nanoelectronic devices because of its large on-state current and tunneling area [25]. However, research concerning its single-event irradiation is very rare. The SEE of TGTFET is studied according to the method of studying the SEE in FDSOI, which has been widely investigated. At the same time, this paper studies the effect of large LET on TGTFET devices, which has not been studied.

In this paper, SEE in TGTFET is studied, and the specific content is as follows. Section 2 shows the basic physical simulation model and simulation methods. Section 3 characterizes the effect of SEE on TGTFET by studying the transient current and collecting charge. Section 4 presents the conclusion. The findings in this paper can be used as a reference for studying the SEE of TFET devices and at the same time provide ideas for finding the sensitive nodes of TFET to SEE.

## 2. Materials and Methods

### 2.1. Simulation Model in TFET

All semiconductor devices in technology computer-aided design are simulated in the same way, with the only difference being the physical model and model parameters used in Sdevice simulation. Due to the fact that the source and the drain in TFET are heavily doped, the carrier statistical model adopts Fermi statistical distribution [26]. A high doping concentration causes a reduction in the bandgaps of the semiconductor materials, such as silicon. Therefore, the bandgap narrowing model is adopted. Multiple types of impurities are introduced into the process of heavy doping of the source and drain in TFET. At the same time, a higher transverse electric field will be generated, and the carriers are easy to collide and scatter when it is transported on the channel surface. The drift velocity of the carriers may also become saturated under the high electric field. Therefore, the mobility model should consider the scattering model of ionized impurities ($\mu_{\mathrm{dop}}$), the interface scattering model ($\mu_{\mathrm{InterSc}}$), and the high-field saturation model ($\mu_{F}$) [27,28,29,30], and the final effective mobility model can be expressed by

(1) $$\frac{1}{\mu }=\frac{1}{\mu_{\mathrm{dop}}}+\frac{1}{\mu_{\mathrm{InterSc}}}+\frac{1}{\mu_{F}}$$

The carrier recombination model adopts the SRH model [31]. There are two types of tunneling models in the Sentaurus software. The local BTBT model assumes that the electric field at the tunneling junction is constant, and the tunneling barrier is equivalent to the triangular barrier, that is, the electric field is constant at every point in the tunneling path. The nonlocal BTBT model considers the electric field at each point in the tunneling path as a variable, which means the BTBT tunneling probability depends on the band bending at the tunneling junction. The nonlocal tunneling model is more in line with the actual situation of TFET simulation [32]. The nonlocal BTBT model is used in this paper.

### 2.2. Simulation Model of SEE

**Figure 1:**
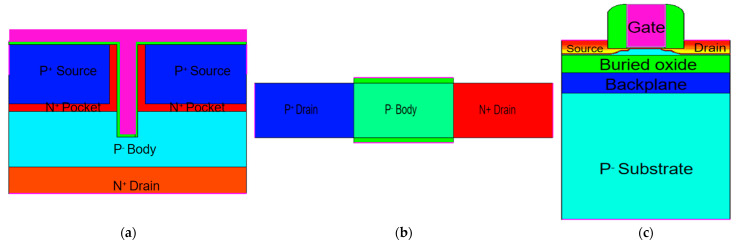
Cross-sectional view of (**a**) TGTFET, (**b**) DGTFET, and (**c**) FDSOI MOSFET considered in this paper.

**Figure 2:**
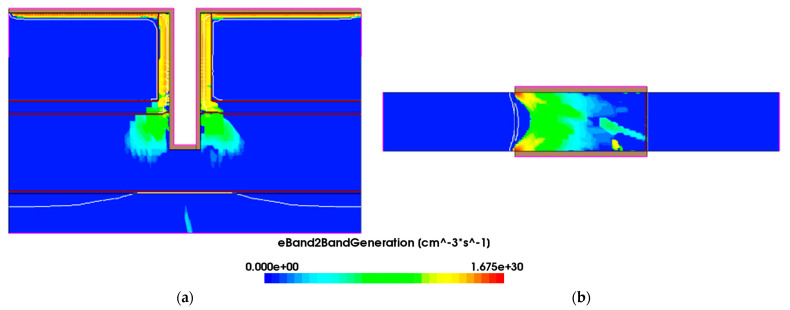
BTBT tunneling generation of (**a**) TGTFET and (**b**) DGTFET at on state.

During this simulation, silicon is used as the source, drain, channel and substrate, and SiO_2_ is used as the BOX layer. HfO_2_ is used as a gate dielectric. Meanwhile, the metal-gate work function is Φ_MS_ = 4.2 eV. Compared with the traditional double gate TFET (DGTFET) structure, TGTFET has a dual-source structure, as shown in Figure 1. The structure can effectively improve the BTBT tunneling probability and the on-state current of the device. The gate covers the middle groove and the top of the source region, enabling the vertical and horizontal direction in which the BTBT tunneling can occur, as shown in Figure 2a, thereby increasing the tunneling probability and improving device performance. The physical and technological parameters of the structure in TGTFET are shown in Table 1.

To perform these comparisons, a DGTFET and a 65-nm ultrathin FDSOI nMOSFET device are created as shown in Figure 1b,c [33]. In the DGTFET, the length of channel is 50 nm, the length of source and drain is 50 nm, the height of source and drain is 20 nm, the drain has an n-type constant doping profile of 1 × 10^18^ cm^−3^, and the source and channel/body have a p-type constant doping profile of 1 × 10^20^ cm^−3^ and 1 × 10^1^^7^ cm^−3^, respectively. In the FDSOI, which is fabricated by lightly doped drain/source (LDD) technology, the length of the spacer is 32.5 nm, the effective length of the gate is 59 nm, the height of source and drain is 44 nm, and the thicknesses of the body and BOX layer are 20 nm and 50 nm, respectively. The source and drain regions have a 1 × 10^20^ cm^−3^ n-type Gaussian doping profile, and the body and substrate of the device have a p-type constant doping profile of 1 × 10^15^ cm^−3^ and 1 × 10^14^ cm^−3^, respectively. There is a back-plane layer (BP) between the body and substrate of the FDSOI, which has a 60-nm thickness and 2 × 10^18^ cm^−3^ constant doping. In this case, the metal-gate work function is Φ_MS_ = 4.2 eV.

**Figure 3:**
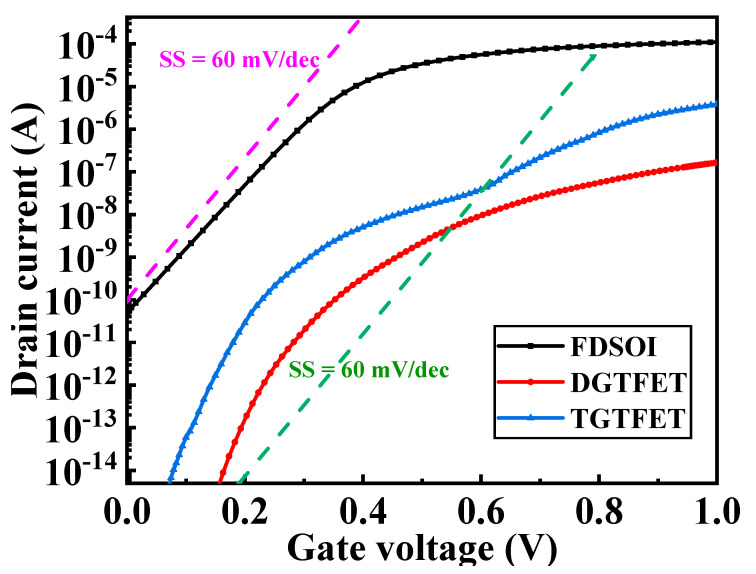
Transfer characteristics for TGTFET, DGTFET, and FDSOI at V_d_ = 0.5 V.

Figure 3 shows the transfer characteristics of the above three devices at 0.5 V drain bias. Compared with planar TFET, TGTFET has higher on-state current due to the dual-source structure. The I_on_/I_off_ in TGTFET reaches 4.17 × 10^9^, while in FDSOI it is 5 × 10^6^. TGTFET exhibits larger drain current fluctuation at a lower gate voltage. Meanwhile, this device exhibits larger SS variation and can achieve an SS smaller than 60 mV/dec, which cannot be obtained in FDSOI devices. Hence, the TGTFET is one of the most promising candidates to replace MOSFET in low-power logic circuit applications.

The effect of ion strike has been simulated by the heavy-ion model in Sdevice (a module in the Sentaurus software used to add physical models). The generation rate caused by the heavy ion is computed by [34]:

(2) $$G(l,w,t)=G_{\mathrm{LET}}(l)R(w,l)T(t)$$

where *R*(*w*) and *T*(*t*) are functions describing the spatial variation of the generation. *G*_LET_(*l*) is the linear energy transfer generation density, and its unit is pairs/cm^3^. In this paper, the depth, radius, and time of the heavy ion incident is set as 0.5 um, 0.1 um, and 5 × 10^−12^ s, respectively. The primary parameters of the heavy-ion irradiation model are shown in Table 2. To investigate the effect of drain voltage on TGTFET SEEs, a different drain voltage bias is used at the same gate voltage and LET. Due to studying the effect of LET on TGTFET SEEs, a charge deposition of 2–10 pC/μm was chosen, approximately equivalent to LET = 200–1000 MeV·cm^2^/mg. To explore the sensitive region in TGTFET, the heavy ions impact the different regions in TGTFET considering the vertical and horizontal direction.

## 3. Results and Discussion

### 3.1. Heavy Ions Sensitive Region

When heavy ions hit the sensitive region of the device, massive e–h pairs will be generated, and the e–h pairs will be collected by the terminal using a drift diffusion mechanism along the incident path. If the amount of collected charge reaches a certain inversion threshold, it will cause the logic state of the circuit to change. At the same time, the collected charge will form a current pulse in the sensitive region, affecting subsequent circuits. Therefore, transient current and collected charge are two important parameters for studying the single-event transient in TGTFET.

**Figure 4:**
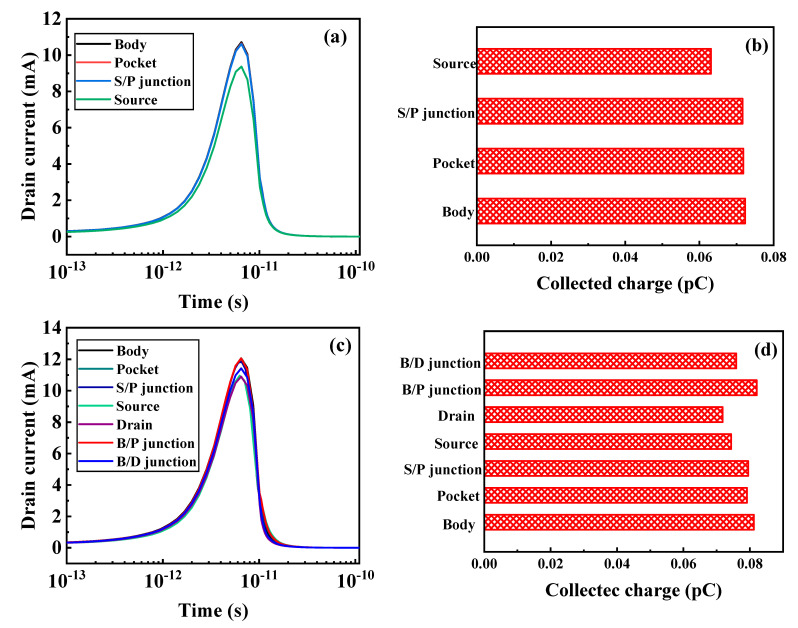
Simulated (**a**) drain transient currents and (**b**) collected charges for four vertical ion hit locations, and (**c**) drain transient currents and (**d**) collected charges for seven horizontal ion hit locations in TGTFET devices at LET = 100 MeV·cm^2^/mg and V_d_ = 0.5 V.

The heavy-ion-sensitive region is the region where the device has the strongest reaction to heavy-ion irradiation and may even cause the device to fail. Finding the sensitive region of the heavy-ion radiation and optimizing and strengthening the sensitive area in advance, the resistance of the device to radiation can improve. To find the sensitive area in the TGTFET, when LET = 100 MeV·cm^2^/mg and V_d_ = 0.5 V, heavy ions are incident in four vertical locations and seven horizontal locations. Figure 4 shows the transient current and collected charge at different hit locations.

**Figure 5:**
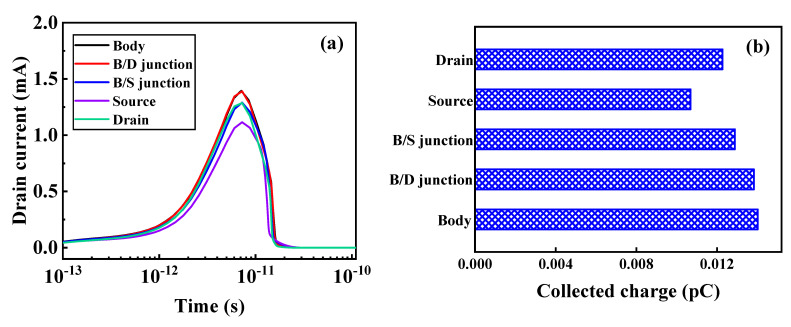

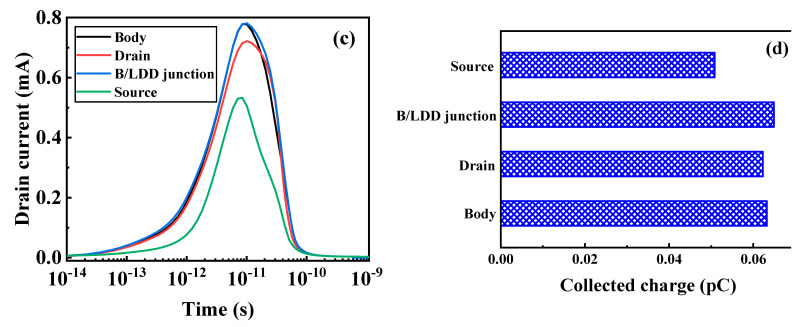
Simulated (**a**) drain transient currents and (**b**) collected charges for five vertical ion hit locations in DGTFET, and (**c**) drain transient currents and (**d**) collected charges for four vertical ion hit locations in FDSOI at LET = 100 MeV·cm^2^/mg and V_d_ = 0.5 V.

As shown in Figure 4a,b, when heavy ions are vertically incident, the transient current and collected charge in the body region, pocket region, and S/P junction (the junction between the source region and pocket region) are relatively large. In Figure 4c,d, when heavy ions are incident horizontally, the transient current and collected charge are the largest in the B/P junction (the junction between the body region and pocket region). In summary, the SEE-sensitive regions in TGTFET are the B/P junction and the S/P junction, which are the tunneling junctions. As shown in Figure 5a,b, when heavy ions are vertically incident, the transient current and collected charge in the body and B/D junction (the junction between the body region and drain region) are large. The SEE-sensitive regions in DGTFET are the body and the B/D junction. In Figure 5c,d, when heavy ions are vertically incident, the transient current and collected charge in the body/LDD junction (the junction between the body region and lightly doped drain region) are large. The SEE-sensitive region in FDSOI is the body/LDD junction.

In TGTFET, the existence of the pocket layer improves the probability of band tunneling. The transient current and collected charge near the body area and the pocket layer are relatively large, as shown in Figure 4. This is the same as the conclusion that the SEE- sensitive region in LTFET is the tunnel junction [24]. However, in the DGTFET, its SEE-sensitive region is not the tunnel junction but the body region and B/D junction. This is because the DGTFET is a horizontal tunneling TFET. After the drain bias is added, the B/D junction is equivalent to a reverse biased PN junction, which will produce a funnel collection effect. Both TGTFET and LTFET are vertical TFETs and have the same tunneling mechanism, so the sensitive area for SEE is the same. FDSOI is a horizontal conduction device, so the sensitive area in FDSOI is the reverse biased PN junction, similar to DGTFET. When designing for radiation resistance, it is necessary to consider the radiation-sensitive area. Therefore, the research in this paper has guiding significance for the design of radiation reinforcement.

It can be seen from Figure 5 that the transient current of FDSOI is lower than that of DGTFET, but the collected charge is much higher than DGTFET. This is because the top silicon film of FDSOI has a thickness of 44 nm, which is greater than the thickness of the DGTFET, and has a large active area, thus collecting a significant amount of charge. Therefore, the use of a thinner active region can effectively reduce the impact of the single event effect.

### 3.2. Impact of Drain Voltage on SEE in TGTFET

**Figure 6:**
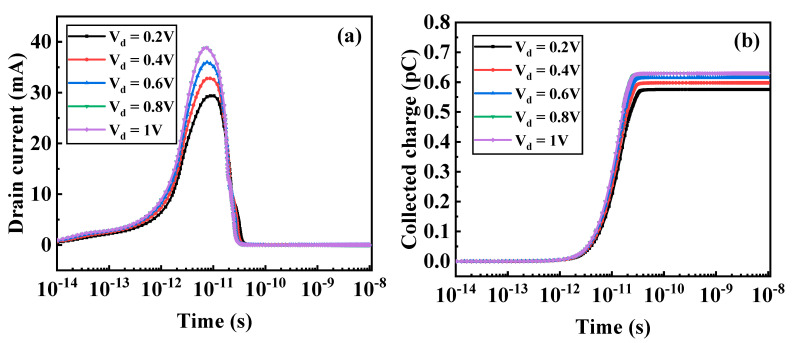
(**a**) Drain transient currents and (**b**) collected charge as a function of time after the ion strike with various drain biases. LET = 1000 MeV·cm^2^/mg.

Figure 6a,b shows the drain current transient and collected charge in TGTFET at different drain biases for an ion striking vertically in the middle of the body. In Figure 6a, as the drain bias increases, the peak value of the transient current also increases accordingly. The recovery time of the transient current is reduced quickly as the drain bias increases. In Figure 6b, the collected charge increases as the drain voltage increases. At the 10^−10^ s, the transient current returns to a minimum and the collected charge tends to saturate.

**Figure 7:**
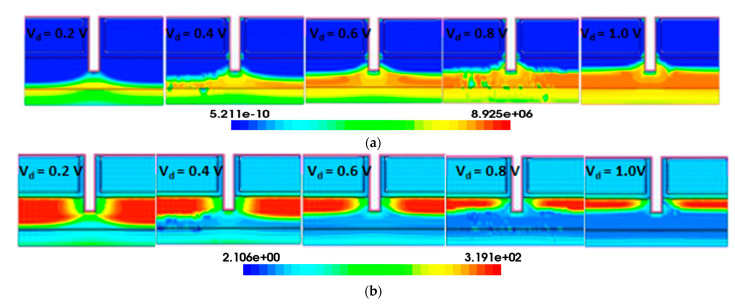
(**a**) Hole velocity and (**b**) hole density in the TGTFET after the ion strike (t = 5 ps). LET = 1000 MeV·cm^2^/mg. V_d_ = 0.2, 0.4, 0.6, 0.8, and 1 V.

Heavy ions are incident vertically from the middle of the body region, and massive e–h pairs are generated. As the drain bias increases, the collision ionization rate in the body region and the drain region of the TGTFET increases, so the carriers generated by the impact ionization also increase. The body and drain regions form a reverse-biased PN junction at V_d_ = 0.5 V. Heavy ions through this reverse-biased PN junction create a funnel-shaped electric field distortion region. The generated electrons will be collected by the drain, and the holes will flow to the body region. As the electrons are collected by the drain, the holes are left in the body region, so the distribution of holes needs to be studied. As shown in Figure 7, after the heavy-ion impacting, with the increase of the drain bias, the density of the holes in the body region decreases, and velocity of the holes increases. Therefore, as the voltage increases, the recovery time of the transient current decreases. Therefore, devices that can work under low drain voltage have better radiation resistance.

### 3.3. Impact of LET on Single-Event Transient Effects in TGTFET

**Figure 8:**
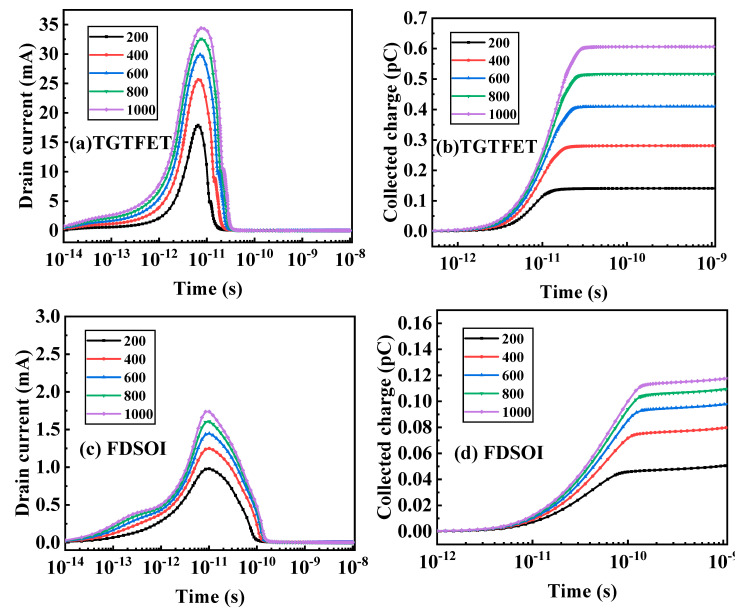
(**a**) Drain transient currents and (**b**) collected charges as a function of time after the ion strike with various LET in TGTFET. (**c**) Drain transient currents and (**d**) collected charges as a function of time after the ion strike with various LET in FDSOI. V_d_ = 0.5 V. LET: MeV·cm^2^/mg.

At different LET, the amount of charge deposition must be different. As shown in Figure 8, to further understand the effect of different LET on single-particle transients, the transient current and collected charge in TGTEFT and FDSOI under different LET were studied at V_d_ = 0.5 V.

As shown in Figure 8, as the LET increases, the peak value of the transient current and the collected charge also increase. Under LET = 1000 MeV·cm^2^/mg, the peak transient current of TGTFET is 3.44 × 10^−2^ A, the peak transient current of FDSOI is 1.738 × 10^−3^ A, the saturation collection charge of TGTFET is 0.61 pC, and the maximum collection charge of FDSOI is 0.11 pC. The transient current peak of TGTFET is twice that of FDSOI, and the maximum charge collection is 5.45 times that of FDSOI. As the top silicon film of FDSOI has a small thickness and a buried oxide layer separates the active region and the substrate, there will be fewer carriers left by heavy ions in the active region, thereby reducing the effect of the single-particle effect on the device. Therefore, TGTFET is more sensitive to heavy-ion impact than FDSOI, and it is easier to cause the logic circuit to flip.

As can be seen in Figure 8b,d, after the time is greater than 10^−10^ s, the collected charge in the TGTFET assumes a saturated state but shows an upward trend in FDSOI. In FDSOI, the source region (emission region), the body region (base region), and the drain region (collector region) form a parasitic bipolar transistor. When heavy ions enter the device body region, excess carriers will be generated along the incident track. The holes in the body region are biased externally. Under the influence of the lower source-body barrier, that is, toward the source region, this forms an injection current from the body region to the source region, and electrons will drift toward the drain, which can be compared to the minority carrier from the emission region to the base region. The injection will stimulate the parasitic transistor to turn on, and the bipolar amplification effect occurs. In TGTFET, the bipolar amplification effect can be eliminated. Therefore, even if the transient current recovers, the bipolar amplification effect will cause the collected charge in the FDSOI to continue to increase.

## 4. Conclusions

In this paper, the transient response of TGTFET to heavy-ion irradiation has been studied by simulation. The behavior of TGTFET under heavy-ion irradiation is different from that of FDSOI. The peak value of the transient current of TGTFET can reach 10.63 mA, which is much larger than the on-state current when V_d_ = 0.5 V. This implies that TGTFET is more sensitive to single-event irradiation than FDSOI. By simulating heavy-ion incidence on different locations in the both the vertical and lateral direction of DGTFET, the most sensitive part of the DGTFET to the influence of heavy ions is identified as the tunnel junction. This research shows that there are many strict problems in the radiation reliability of TFETs, which must be considered in the device structure and radiation-hardening design.

## Figures and Tables

**Table 1:** Device parameters used for the simulation.

Parameter Name	Symbol	Value	Unit
Pocket thickness	t_p_	5	nm
Gate oxide thickness	t_ox_	2	nm
Source height	H_S_	40	nm
Drain height	H_D_	18	nm
Body doping	N_B_	1 × 10^17^	cm^−3^
Pocket doping	N_P_	1 × 10^19^	cm^−3^
Source doping	N_S_	1 × 10^20^	cm^−3^
Drain doping	N_D_	1 × 10^18^	cm^−3^

**Table 2:** Heavy-ion model parameters used for the simulation.

Parameters	Value
depth	0.5 um
time	5 × 10^−12^ s
direction	(0,1,0)
location	(0,0,0)
radius	0.1 um
LET	2~10 pC/μm
V_d_	0.2~1 V
